# A Toxic Sterolysin From a 1950s Culture of Gymnodinium Veneficum Ballantine

**DOI:** 10.21203/rs.3.rs-3970188/v1

**Published:** 2024-03-25

**Authors:** Allen R. Place, Josefina Ramos-Franco, Amanda L. Waters, Mark T. Hamann

**Affiliations:** University of Maryland Center for Environmental Sciences; Rush University Medical Center; University of Central Oklahoma; Medical University of South Carolina

**Keywords:** Karlotoxins, Membrane Pores, Sterol Binding

## Abstract

In 1957 Abbott and Ballentine described a highly toxic activity from a dinoflagellate isolated from the English Channel. in 1949 by Mary Park. From a culture maintained at Plymouth Laboratory since 1950, we have been able to isolate two toxic molecules (Abbotoxin and 59-E-Chloro-Abbotoxin), determine the planar structures by analysis of HRMS and 1D and 2D NMR spectra and found them to be karlotoxin (KmTx) congeners. Both toxins kill larval zebrafish with symptoms identical to that described by Abbot and Ballantine for gobies (*Gobius virescens*). Using surface plasma resonance the sterol binding specificity of karlotoxins is shown to require desmethyl sterols. Our results with black lipid membranes indicate that karlotoxin forms large-conductance channels in the lipid membrane, which are characterized by large ionic conductance, poor ionic selectivity, and a complex gating behavior that exhibits strong voltage dependence and multiple gating patterns. In addition, we show that KmTx 2 pore formation is a highly targeted mechanism involving sterol-specificity. This is the first report of the functional properties of the membrane pores formed by karlotoxins and are consistent with the intial observations of Abbott and Ballentine from 1957.

## Introduction

Looking for a good oyster feed, Dr. Mary Parke at the Plymouth Laboratory of the Marine Biological Association isolated a small dinoflagellate from the region near Plymouth Sound (lat. N. 50° 19’30”, long. W 04°10’) on June 8th, 1949. A second very similar dinoflagellate was isolated from the Hamoaze, over Rubble Bank, off King William Point, South Yard, Devonport (lat. N. 50° 21’50”, long. W 04°10’55”) the following year. Both were deposited in the Type Culture Collection (Plymouth collection no 102 and 103, respectively). The two species were described and named, *Gymnodinium vitiligo* and *Gymnodinium veneficum* ^1^. The only difference between the two species was toxicity, as *G. vitiligo* was harmless, whereas *G. veneficum* produced a very powerful toxin which was lethal to fish and nearly every other organism tested including mice.

In 1957, B.C. Abbot and D. Ballantine described the partial purification and characterization of the toxin from *G. veneficum* ^2^. To follow potency of their preparations they developed a fish bioassay with gobies (*G. virescens* and *G. niger*) in which the amount of toxin per ml which caused permanent loss of balance in 8–15 minutes and death in 10–20 minutes was considered 1 unit. A strongly toxic culture was 6 units per ml. In amazing forsight, they found adding an ethanol suspension of cholesterol (0.2 mg/ml) to the bioassy completely inhibited death for a 4 unit exposure to gobies.

**Figure 1:**
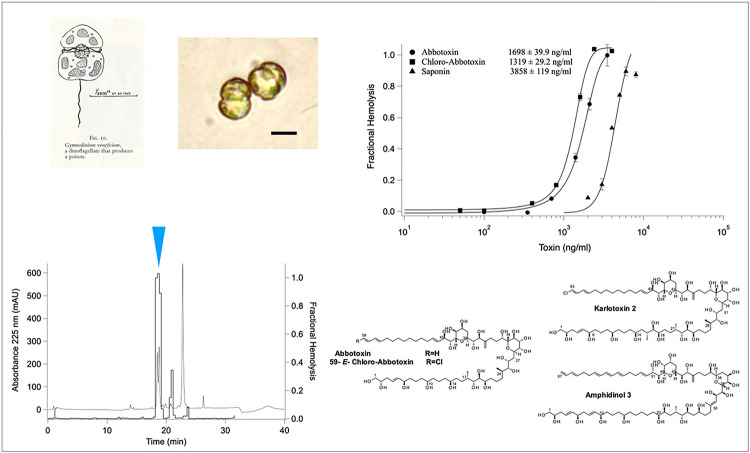
**A.** Left is the original drawing from Nature Adrift: the story of Marine Plankton. Right is photomicrograph of *K. veneficum* Ply 103. **B**. Reverse Phase HPLC Chromatogram of the Hemolytic Fractions (Blue Arrrow). **C**. Hemolytic Activity of purified Abbotoxin and Chloro-Abbotoxin to Rainbow Trout Red Blood Cells. **D**. Structure of Abbotoxin and 59E-Chloro-Abbotoxin compared to KmTx2 and Amphidinol 3

The classic 1962 “*Nature Adrift* - *The Story of Marine Plankton*” by James Fraser ^1^ stated “A poisonous dinoflagellate lives in British waters: It is *Gymnodinium veneficum*, which has been grown in culture at the Marine Biological Station at Plymouth (Fig. 1A).” From the work of B.C. Abbott and Dorrothy Ballantine ^2^ who characterized the toxin activity of *G. veneficum*, concluded that the mode of action was “due to membrane depolarizaton.”^3^. A footnote added reads - “*The conclusions of this paper are of necessity preliminary and need verifying with a purified sample of the toxin*.” We provide this verification in the current manuscript where we present the structure, its mode of action, and sterol specificity.

## Results

Based on all of our analysis (cell size and volume, pigments, sterols, fatty acids and ITS sequence), *Gymnodinium veneficum* (Fig 1A) is a *Karlodinium* species (hereafter referred to as *K. veneficum* PLY 103; ^5^). Ply 103 was still toxic to mussels despite being in culture for 50 years ^6^. Moreover, like Abbot and Ballantine ^2^, we find that 85 to 90% of toxic activity is released upon filtration and can be purified exactly as previously described for karlotoxins ^7^ (Fig 1B). When still in culture at the Plymouth Marine Laboratory, the strain was grown in NaH^13^CO_4_ and sent to Maryland where the compounds were isolated (Fig 1B). Hemolytic activity was used in guided bioassay fractionation and purification. Two structures were elucidated using similar techniques as those previously described ^8^, and all NMR spectra, data tables and key HMBC and COSY correlations and the deletion of carbon from KmTx2 for the final assembly of C1–18 of Abbotoxin are shown in the ***Supporting Information S1–18***. K. veneficum PLY 103 produces two karlotoxins: **Abbotoxin** and **chloro-Abbotoxin**, which are hemolytic to rainbow trout erythrocytes (Fig 1C) and found in nearly equivalent cell quotas (0.93 pg/cell vs 1.25 pg/cell), which is a relatively high cellular toxin quota among known *K veneficum* strains ^9^. These structures are the smallest congeners elucidated to date and differ significantly from KmTx2 in the C1-C18 region which can be seen in Fig. 1D^8^. Abbotoxin and 59E-chloro-Abbotoxin differ from each other only by the absence of a chlorine on the terminal diene of 59E chloro-Abbotoxin. The structure was five carbons shorter compared to KmTx2 all of which come from the polyol region. The HSQC overlay experiments showed that C23-C63 and C65–67 for KmTx2 (**1**) are identical to C19–49 and C60–62 for Abbotoxin. The difference in the structures was the loss of C64, C13–14 and C16–17 (3 CH’s, 1 CH_2_ and 1 CH_3_ [68 amu]) from KmTx2 (***Supporting Information S1–18***).

**Figure 2:**
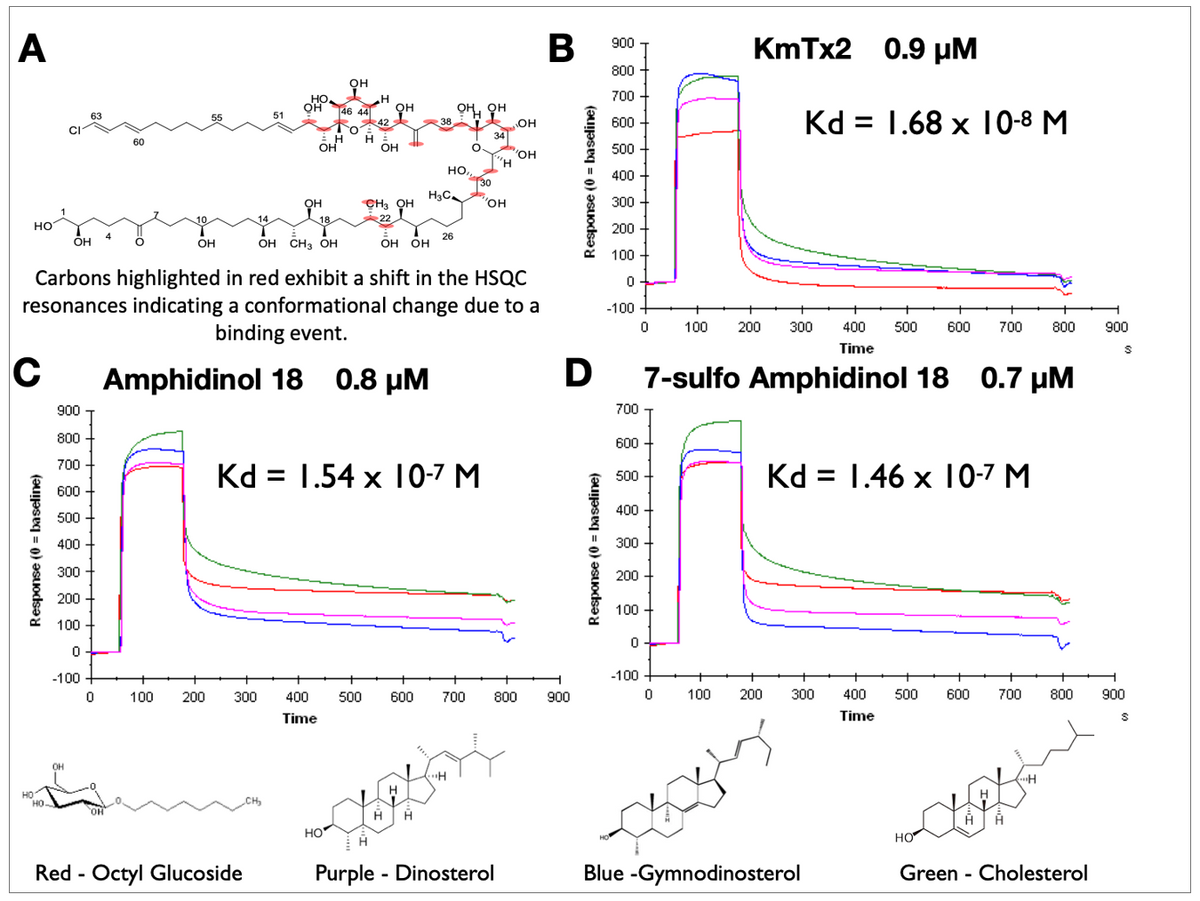
**A.** HSQC resonance shifts when KmTx is in the presence of cholesterol (***Supporting Information S19***). **B**. Sensogram of KmTx2 binding to sterols. **C**. Sensogram of Amphidinol 18 binding to sterols. **D.** Sensogram of 7-Sulfo Amphidinol 18 binding to sterols.

Given the ability of cholesterol to rescue gobies from death ^2^, we showed that some sterols could also inhibit hemolysis by KmTxs in a concentration dependent manner ^10^. Moreover, HSQC overlay studies into the sterol-binding interactions of KmTx with a series of increasing ratios of cholesterol found only certain atoms ^8^ were perturbed (Fig 2A). There were definitive shifts for certain carbon atoms, mainly around the tetrahydropyran ring systems, while other carbon atoms (e.g., C1) on the exterior of the structure were untouched by the addition of cholesterol. Based on the cholesterol interactions, the molecule appears to prefer a hairpin formation, which has not been seen when the molecule is freely rotating without other molecular interactions.

**Figure 3:**
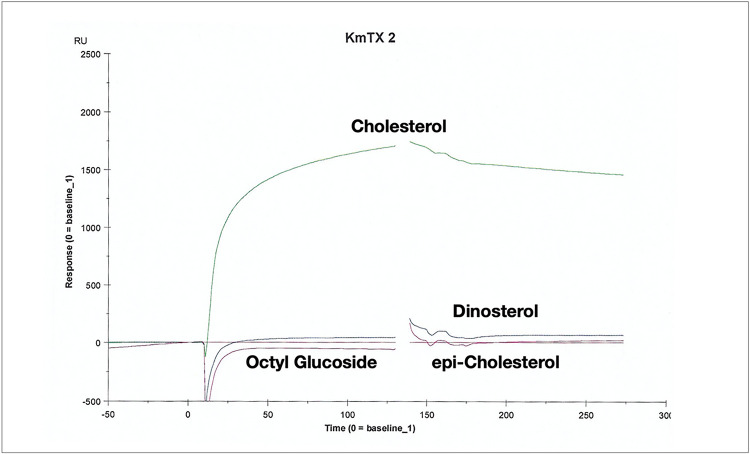
Sensograms for interaction of 10 μM KmTx2 to Series S sensor Chip HPA coated with equivalent amounts of cholesterol, dinosterol and epi-chloresterol and washed with 40 mM octyl glucoside

The dominant sterol of *K. veneficum* is gymnodinosterol (71–83%)^11^ which we find is true with Ply 103. To examine the sterol specificty we performed surface plasmon resonance^12^ with surfaces coated with different sterols (octyl glucoside, dinosterol, gymnodinosterol and cholesterol) interacting with three different sterolysins KmTx2, amphidinol 18, 7-sulfo amphinidol 18). As clear from Figure 2B–D all the sterolysins rapidly (k_a_ ranging from 10^8^ to 10^9^ M^−1^S^−1^) bind hydrophobic and sterol saturated surfaces with dissociation rates (k_d_) differing by orders of magnitude among the surfaces. In general, the k_d_ for cholesterol surfaces (green trace) is slowest for all three sterolysins (***Supporting Information S20***). The stability of the interaction (Fig. 2B–D) is much greater with desmethyl sterols with a 3 beta hydroxyl group. The dissociation constant K_D_ to cholesterol is presented in the insert for each sensorgram. The stability of the interaction with cholesterol is exemplified when bound KmTx2 is washed with the detergent octyl glucoside (Fig. 3) compared to dinosterol and epi-cholesterol.

Single-cell microfluorimetric measurements with fluorescent ion indicators using five differentiated mammalian cell types, including rat embryo fibroblasts, human T-lymphocytes, rat intestinal epithelial cells, rabbit vagal sensory neurons, and rat ventricular cardiac myocytes, showed that KmTx2 causes a pre-lytic increase in the permeability of the plasma membrane to cations such as Na^+^, Ca^2+^, and Mn^2+ 13^. Despite the information provided there and previously suggesting that karlotoxins non-specifically increase permeability in vertebrate (cholesterol containing) membranes leading to colloid osmotic lysis ^10^, the structure of the pore itself has yet to be determined. To this end, we reconstituted KmTx2 into artificial planar bilayers to investigate whether or not they can form membrane pores in the absence of proteins and if so, to define the biophysical profile of these pores in terms of their permeability and conductance.

**Figure 4:**
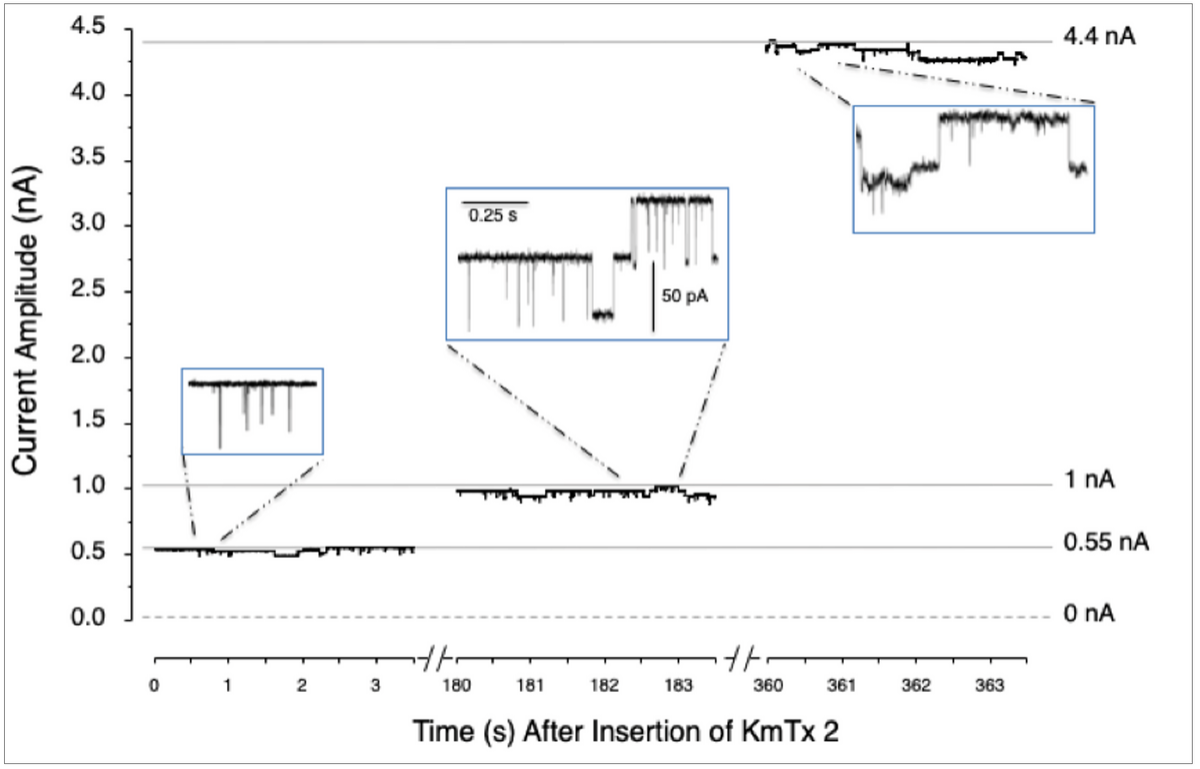
KmTx2 Pore-forming Activity. Representative single-channel events recorded at the indicated times during a 6 minutes period following the addition of the toxin. The toxin concentration was 100 ng/ml added to a DOPE:DOPS:DOPC and Cholesterol bilayer bathed in KCl gradient (500 mM/100 mM) and held at +100 mV. Insets show expanded time scales of three sections showing the single-channel opening and closing transitions with a unitary conductance of ~500 pS.

Addition of the toxin to one side of the bilayer resulted in well defined current fluctuations between the close state (zero-current level) and a single conducting, main open state. The current level transitions, characteristic of the single-channel activity, were observed at both positive and negative potentials. Using KCl solutions we observed the channel conductance increased gradually, reaching a steady-state level within 3–8 min after the toxin insertion to the bilayer (100 ng/ml). This behavior is illustrated in Fig. 4, where a series of current records were taken at the marked time, in a period of 6 s. Open state is shown as upward current deflections in response of a voltage of +100 mV. In the presence of a KCl gradient (500/100 mM), about 90% of the channels had a conductance of 560 ± 0.49 pS (n=5) in the first minutes of their incorporation into the lipid bilayer. After ~10 minutes the main conductance at positive potentials increased to 17.9 ± 1.35 nS (n=3).

**Figure 5:**
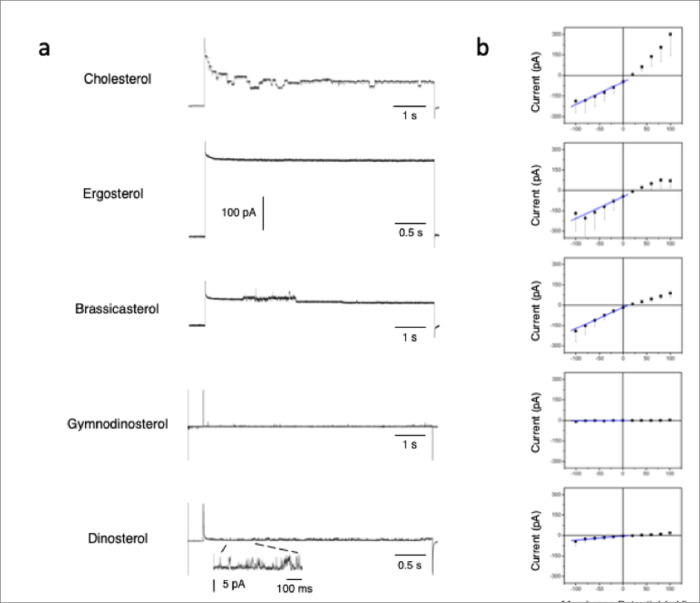
KmTx2 pore-forming Activity is Influence by the Lipid Composition of the Bilayer. a. Single channel traces of KmTx 2 incorporated in lipid bilayers made out of different types of lipids (DOPE:DOPS:DOPC:”X”; 4.7:2.8:1.9:0.6 ratios). b. KmTx2 channel mean current amplitude (pA; black squares) plotted as a function of the membrane potential (mV) for the different tested lipids. Conductance was determined by linear regression (blue line) of the current values from −100 to 0 mV, in asymmetrical solutions (100/20 mM KCl cis/trans). Data are shown as means ± S.E.M. of 2–7 measurements.

To corroborate the toxin’s sterol specificity we determine the lipid selectivity of the channel/pore formation. Thus, unitary current was recorded in phospholipid bilayers containing different sterols. The traces presented in Fig. 5A show the unitary current recorded after KmTx2 insertion in the presence of a KCl gradient (100/20 mM; *cis/trans*) and during the application of a voltage pulse from 0 to +100 mV. Under these conditions, it can be observed that the mean current level (taken as an indication of the level of toxin incorporation) greatly varies depending on the sterol composition of the lipid bilayer. In general, two types of incorporation were observed. In one, the mean current level was very substantial (>100 pA) in bilayers made out of cholesterol, ergosterol, and brassicasterol. A second type of toxin incorporation was observed in bilayers composed by gymnodinosterol and dinosterol, where the incorporation was negligible as indicated by the small current level. These types of behavior are more evident in the current-voltage (I-V) curves shown to the right of the traces, constructed from 4 bilayers for each condition (Fig 5B). The first three conditions exhibit robust current values of >150 pA at extreme potentials (±100 mV). In contrast, the two last conditions resulted in either no channel formation or the reconstituted channel activity was characterized by opening events of small amplitude (~4 pA). These events also exhibited short open and close times (see expanded trace) compared to the toxin’s gating behavior when it was inserted into bilayers containing cholesterol (first trace). In addition, in cholesterol-containing bilayers, KmTx exhibits a constant conductance value, while in ergosterol and brassicasterol, the conductance shows a clear rectification (i.e. reduction) at positive potentials. This behavior could result from asymmetric permeability properties of the toxin when is incorporated into bilayers containing different sterols. To quantify the levels of incorporation in different sterol composition bilayers, the ionic conductance was measured at negative potentials where the conductance remained more constant. The following values of ionic conductance (in nS; n = 7; mean ± SEM) were obtained: cholesterol 1.65 ± 0.6, ergosterol 1.58 ± 1.0, brassicasterol 1.56 ± 0.4, gymnodinosterol 0.02 ± 0.1, dinosterol 0.29 ± 0.18. Conductance was determined by linear regression of the current values measured between −100 to 0 mV, in asymmetrical solutions (100/20 mM KCl cis/trans for cholesterol, ergosterol and brassicasterol and 400/20 KCl gradient for brassicasterol and gymnodinosterol).

Another apparent effect induced by the different sterols used in our studies was the time for the toxin’s incorporation to the bilayer. In the presence of cholesterol, this occurred during the first 10 min; with brassicasterol and ergosterol incorporations events took up to an hour. With the other tested lipids gymnosterol and dinosterol, few or no incorporations were observed up to 4 hours of incubation. These results have been observed even when the K+ concentration was increased up to 500 mM to enhance the likelihood of incorporation into the bilayer (normally, the toxin incorporates in 100 mM K+). In all these experiments the toxin concentration varied between 100–400 ng/ml.

## Discussion

Since the original isolation ^14^ of the polyhydroxypolyene molecule, amphidinol 1, its hemolytic and antifungal activity was apparent. The subsequent seven congeners ^15,16^ all exhibited antifungal, hemolytic, cytotoxic and icththyotoxic activities and a strong surfactant property with the ability to perturb membranes. The sterol dependency of this membrane disruption was further shown with calcein leakage from liposomes containing cholesterol ^17^ and that the two tetrahydropyran rings (which are antipodal ^18^) take a hairpin conformation ^19^ permitting insertion into the bilayer. Further, that amphidinol interacts with membrane sterols through the strict molecular recognition of the stereochemistry of the sterol 3-OH group ^20^. Finally, recent lipid bilayer studies have shown amphidinol 3 forms different types of sterol-aided channels in a concentration dependent manner ^21,22^.

Here, all three sterolysins (KmTx2, AM18, and 7-sulfo-AM18) bind quickly to sterol surfaces with 1:1 stoichometry but exhibited slow off rates to cholesterol (Fig. 2, ***Supporting Information S20***). The binding is sufficiently stable that washing the surface with 40 mM octyl glucoside has little effect (Fig. 3). The importance of the 3-OH stereochemistry as well as the sterol needubgto be a des-methyl is clear. Karlotoxins exhibit all these properties and more. We find that the mechanism of KmTx2 toxicity seems to be through formation of membrane pores that affect membrane integrity, promoting ionic gradient dissipation and facilitating internalization of other toxic molecules. Until now, there have been no reports of KmTx-formed ion channels, but the data reported here. In our lipid bilayer studies, every time the toxin was added to the Cis side of the chamber, channel-like activity was observed. This activity was characterized by current transitions from the baseline resembling the common ion-channel activity. In addition, the channel conductance increased with time and this was most likely the result of further channel insertions because the cis chamber was not washed out to remove the toxin. The observed channel conductance varied from picosiemens to nanosiemens during the time of the experimental recording. Another interesting feature exhibited by the toxin was conductance rectification observed at positive potentials, while at negative potentials (0 to −100 mV) the conductance showed ohmic (i.e. linear) behavior. This behavior could result from asymmetric permeability properties of the toxin when is incorporated into bilayers containing different sterols.

We found that KmTx2 toxicity is a highly targeted mechanism that involves special lipid specificity for the pore formation. In lipid bilayers containing gymnodinosterol or dinosterol (the sterols used by dinoflagellates) KmTx2 failed to form pores. In those conditions increasing the KCl gradient and toxin concentration failed to favor toxin insertions. This toxin sterol-specificity has been documented for other toxins like Amphotericin B ^23^.

Another important finding was that KmTx2 forms a pore permeable to K+. The KmTx2 ion channel cationic nature was confirmed by the reversal potential (Vrev) of the I-V relationship being toward the equilibrium potential for K+ (EK). This finding suggests that pores formed by KmTx2 displays a preferentially cationic selectivity. The ***Supporting Information S21*** shows the summary of K+ permeation experiments. Here the Vrev was measured at different K+ concentrations (in the trans chamber) and with bilayers built with different types of lipids. The ionic conditions in the cis chamber remained constant (100 mM KCl) throughout these experiments. With 100 mM M KCl on one side of the membrane and 20 mM KCI on the other, we measured an average Vrev of +34 mV, indicating cationic selectivity (close to the K+ equilibrium potential (EK) +40 mV). Similar results were obtained with different types of lipids which did not modify the pore’s selectivity for K+, as indicated by its dependence on the trans K+ concentration. As mentioned above, Abbott and Ballantine results showed that *Gymnodinium* toxin induced a substantial reduction of muscle excitability, likely due to an extensive membrane depolarization ^2^. Consistent with our results, the dramatic effects induced by the toxin can be explained by formation of poorly selective membrane ion channels that will enable a quick dissipation of [Na+] and [K+] gradients. The consequent reduction in the equilibrium potential for these ions will result in a substantial membrane depolarization with the subsequent reduction in cell excitability.

Findings from our study point to the involvement of a specific sterol recognition toxin site(s). We found that KmTx2 toxicity is a highly targeted mechanism that involves special lipid specificity for the pore formation. In lipid bilayers containing gymnodinosterol or dinosterol (the sterols used by dinoflagellates) KmTx2 failed to form pores. In those conditions increasing the KCl gradient and toxin concentration failed to favor toxin insertions. This toxin sterol-specificity has been documented for other toxins like Amphotericin B ^24^ cytolysin A ^25^ and in the dinoflagellate *Alexandrium tamarense* ^26^.

Dinoflagellates make a diverse repertoire of natural products ^27,28^ by unique biosynthetic pathways.^29^ The ecological *raison d’etre* for these natural products is largely unknown, except for the sterolysins. For *K. veneficum*, it provides the means for prey capture ^30^ and predator avoidance. ^31,32^ By having a desmethyl sterol targeted pore forming toxin each dinoflagellate species ensures it will not self-intoxicate.

## CONCLUSIONS

1. The original toxin described in 1957 is a karlotoxin congener.
2. Surface plasmon resonance measurements to sterol coated surfaces indicates a rapid binding to desmethyl sterols with a 3β hydroxyl specificy.
3. The planar lipid bilayer data demonstrate that KmTx2 has intrinsic pore-forming activity.
4. KmTx2 channels exhibited a preference for cations over anions, demonstrated by the reversal potential.
5. KmTx2 pore formation is sterol-specific
6. K^+^ selectivity of the KmTx2 pore is not affected by the different lipids tested.

## Material and Methods

### Culturing and Strains.

*K. veneficum* strain CCMP 2064 that produces Kmtx2 was acquired from the Center for the Culture of Marine Phytoplankton (CCMP). CCMP 2064 was isolated in November 1998 from a fish-kill on the Wilmington River, Georgia and deposited on May 3, 2003. CCMP 2064 is unialgal but not axenic and was cultured phototrophically in 15 psu filtered (0.22 μm) natural seawater combined with f/2 nutrients and vitamins without Si^−1^ at 100 Einstein m^−2^s^−1^ and 20°C ambient temperature. Cell densities were measured using a Coulter Multisizer II (Beckman-Coulter) counter and cells mL^−1^ determined using Accucomp (Version 2.01) software. Abbotoxin and 59-E-chloro-Abbotoxin were isolated from the type species (PLY 103) for *K. veneficum* originally isolated in 1950 from Plymouth Sound, UK (50.364 N, 04.182 W). PLY 103 was grown in 500 ml glass flasks with 300 ml of culture in ERD-SCHREIBER medium at 15 degrees and 12:12 light:dark cycle. ambient temperature. Richard Pipe of the MBA initially grew 3 liters of PLY 103 (~19, 700 cells/ml; ~6 × 10^7^ cells). The cultures were filtered on GF/F filters and the filters and frozen filtrate on dry ice were sent to Maryland. Subsequently, he grew 14 liters of PLY 103 grown in Erd-Schreiber Media containing 50 mg NaH^13^CO_4_ (sodium bicarbonate) and placed the filtrate on a 55g C18 columns (AnaLogix, Sorbtech Technologies) for shipping to Maryland.

### Sterol Isolation^10^.

Ergosterol, cholesterol and epicholesterol were obtained from Avanti Polar Lipids (Alabaster, AL). For dinoflagellate sterols, cultures of the KmTx2-producing *K. veneficum* isolate CCMP 2282, grown as described above were filtered onto glass-fibre filters and extracted twice with chloroform/methanol (2:1, v/v). Neutral lipids were isolated using activated silica according to Yongmanitchai and Ward ^33^. Neutral lipids were further separated using thin-layer chromatography (TLC). Individual bands were scraped and the sterol-containing fraction was confirmed by thin-layer chromatography-flame ionization detection (TLC-FID) using an IATROSCAN TH-10 TLC-FID Analyzer (Iatro Laboratories, Tokyo, Japan). The sterol containing band was further separated by reversed-phase high-performance liquid chromatography (HPLC). Sterol peaks were positively identified by gas chromatography mass spectrometry (GC-MS) according to Leblond and Chapman ^34^. The fraction containing (24S)-4α-methyl-5α-ergosta-8(14),22-dien-3β-ol (gymnodinosterol) was determined to be >95% pure. Dinosterol and amphisterol was purified similarly from *Crypthecodinium cohnii* (ATCC 30334) *and Amphidinium carterae* (CCMP 1314), respectively. Each were determined to be > 95% pure by GC-MS.

### Toxin Isolation.

The KmTx2 standard was isolated by the following procedure ^7^. To obtain adequate quantities of metabolite for structural characterization, 5.8 ×10^9^ cells (40 L of culture) were grown with NaH^13^CO_3_ (50 mg/L) for 28 days, filtered onto 125 mm GF/F filters (Whatman), and the metabolite (>95% of recovered material) from the filtrate was concentrated on three 55 g C18 columns (AnaLogix, Sorbtech Technologies) in parallel. Extraction of the cells with MeOH only provided 5% of the recovered metabolite. The columns had been activated by passing 200 mL MeOH followed by 200 mL HPLC-grade water through each column. Metabolite was eluted using 200 mL MeOH:water starting at 100% water and continuing in 20% increments to 100% MeOH. The 60% and 80% MeOH fractions were collected, pooled and dried under vacuum at 40 °C. The concentrated metabolite from the pooled 60–80% C18 eluent was then purified using reverse phase chromatography on a semi-preparative scale C18 column (Phenomenex Hyperprep HS C18–80S, 250 × 10 mm, 5 μ), at a flow rate of 4 mL/min. Fractions corresponding to KmTx2 were collected using a fraction collector (61364A, AFC, Agilent) based on the known retention times. Fractions with the same elution times were pooled and dried under vacuum and re-suspended in MeOH:water (80:20). Because of co-elution of congeners, each pooled fraction was further separated on normal phase chromatography as described ^7^ to provide pure KmTx2.

To purify Abbotoxin and 59-E-chloro-Abbotoxin the same procedure as described above was performed. Extraction of the cells with MeOH only provided 5% of the recovered metabolite. From the 60–80% methanol fraction off the C18 flash columns two major fractions could be obtained from the semi-preparative C18 column (Figure 1B). The two active fractions contained co-elution of a 58 dalton congener (~ 1 to 5%) which required each pooled fraction to be further separated on normal phase chromatography as described ^7^. From the 14 liters of PLY 103 2 mg of Abbotoxin and 2.1 mg 59-E-chloro-Abbotoxin were purified. The C13 enrichment was estimated to be about 10%.

**Amphidinol 18** ([M+Na]+ m/z] 1381.82575) and its **7-sulfate** ([M − H + 2Na (calcd for C71H121Na2O27S, 1483.76059) ^35^ derivative was isolated from *Amphidinium carterae* (CCMP 1314). The dinoflagellate was cultured in L1 medium at 20.0 ±0.5 °C, under a 14:10 light/dark regime and at 100 μmol m-2s-1. The cells were mass-cultivated in a 40 liter container with pH (Ph 8.50) control through addition of CO2. The initial cell density was around 8000 cells/mL. During the exponential phase, media was refreshed with 4 L additions of L1 medium. In the stationary phase (final cell density: 480 000 cells/mL), the 40 L of culture was harvested in a swing-out centrifuge, for 10 min at 4 °C at 2300 g. The cell pellet (9.8 g) was stored at −80 °C until analysis.

The cell pellet of was extracted sequentially with 250 mL of CH_2_CL_2_/MeOH (1:2, 1:1, 2:1); the combined extracts were added to a 2 L separatory funnel, and water added to obtain two phases. The lower organic phase was removed and saved for lipid and sterol isolation. The upper phase contained greater than 95% of the amphidinols. After dilution tenfold with water, the extract was placed on a 55 g C18 column (AnaLogix, Sorbtech Technologies) and fractionated as described above for karlotoxins.

### Sterolysin-Sterol Interactions by Surface Plasma Resonance.

By using surface plasmon resonance (SPR) techniques, which are shown to be useful for membrane-bound peptides and drugs, we successfully evaluated interaction between various sterolysins and sterol surfaces. Toxin binding interactions with various lipids were investigated using Biacore T200 and Series S Sensor Chip HPA. The HPA chip surface is composed of alkanethiol (C11) to facilitate adsorption of a lipid monolayer. The HPA chip was prepared according to the instructions for use. The running buffer used was HBS (10 mM HEPES, 150 mM NaCl, pH 7.4). The HPA chip was first pre-conditioned by injection of 40 mM octyl D-glucoside for 5 minutes at 10 μL/minute. Cholesterol, Dinosterol, and epi-Cholesterol were each captured to approximately 1000 RU onto flow cells 2, 3, and 4 respectively using a 30 minute injection at 2 μL/minute. Flow cell 1 containing octyl D-glucoside served as a negative control and reference. Each of the lipid surfaces were washed using a 30 second injection of 10 mM NaOH to stabilize the baseline, and then blocked using 5 minute injection of 0.1 mg/mL BSA. Toxin binding specificity was measured using 1–10 μM KmTx 2 injected over all four flow cells at 10 μL/minute. Reference subtracted sensorgrams are shown. Detection of sterolysin affinity was carried out in HEPES buffer (pH 7.4) as running and injection media. Sterolysin dissolved in HEPES buffer was then passed on the sensor chip treated with sterols. The SPR response increased immediately after injection due to interaction between sterolysin in the sample solution and sterols immobilized on the surface of the sensor chip. To evaluate sterolysin binding to sterol-containing or sterol-free surface, the SPR response in the control lane was subtracted from that in the sterol-captured lane. Sterolysins firmly interacted with the sensor-chip surface and the sterolysins could be washed off. Toxin binding interactions with various lipids were investigated using Biacore T200 and Series S Sensor Chip HPA. The HPA chip surface is composed of alkanethiol to facilitate adsorption of a lipid monolayer. The HPA chip was prepared according to the instructions for use. The running buffer used was HBS (10 mM HEPES, 150 mM NaCl, pH 7.4). The HPA chip was first pre-conditioned by injection of 40 mM octyl D-glucoside for 5 minutes at 10 μL/minute. Cholesterol, Dinosterol, and epi-Cholesterol were each captured to approximately 1000 RU onto flow cells 2, 3, and 4 respectively using a 30-minute injection at 2 μL/minute. Flow cell 1 containing octyl D-glucoside served as a negative control and reference. Each of the lipid surfaces were washed using a 30 second injection of 10 mM NaOH to stabilize the baseline, and then blocked using 5-minute injection of 0.1 mg/mL BSA. Toxin binding specificity, as well as approximate kinetics and affinity were measured using a single concentration of 1.5 μM toxin injected over all four flow cells at 10 μL/minute. Reference subtracted and blank subtracted binding responses were fitted using a 1:1 model, with responses in blue and fitted curves in black.

### Toxin reconstitution into artificial lipid planar bilayers.

Planar lipid bilayers were formed across a 150 μm-diameter aperture in a Delrin partition as described elsewhere ^36^. Lipid bilayer-forming solution contained a 4.7:2.8:1.9:0.6 mixture of 1,2-dioleoyl-sn-glycero-3-phosphoethanolamine (DOPE): 1,2-dioleoyl-sn-glycero-3-phospho-L-serine (DOPS): 1,2-dioleoyl-sn-glycero-3-phosphocholine (DOPC): sterol (1 of 5, cholesterol, ergosterol, brassicasterol, dinosterol and gymnodinosterol) (Avanti Polar Lipids, Alabaster, AL), dissolved in decane (50 mg/ml). KmTx 2 100–200 ng/ml was added to the solution in one side of the bilayer (defined as the Cis chamber; virtual ground). The membrane potential was held in the other side, defined as the Trans chamber. Standard solutions contained KCl (at the indicated concentrations) and 5 mM HEPES (pH 7.35). To record single-channel activity, the Cis and Trans chambers were connected via Ag/AgCl electrodes in series to the head stage of an Axopatch 200B amplifier in the voltage-clamp mode. Unitary current was recorded with commercially available acquisition software (pClamp-9; Molecular Devices, LLC, Sunnyvale, CA) and a 16-bit A/D-D/A converter (Digidata 1322A, Axon Instruments) controlled by a 32-bit PC. Unitary current was digitized at 5 kHz and filtered at 2 kHz. Data analysis was conducted with the analysis package of the same acquisition software (pClamp-9, Molecular Devices, LLC, Sunnyvale, CA).

### General Structural Elucidation Experiments^38^.

^1^H, ^13^C, DEPT, COSY, HSQC, HMBC, NOESY, and ROESY spectra of all congeners were measured on a Bruker Avance or a Varian INOVA 600 MHz NMR equipped with a 3 mm probe.

### NMR Processing Procedures.

Comprehensive NMR data sets were collected for every karltotoxin sample available. Individual experiments were then compared to the known KmTx2 standard data set. The methodologies used here have been applied successfully with other marine molecules such as corozalic acid, an okadaic acid biosynthetic precursor, for quick and efficient structural elucidation^37^. Utilizing the NMR processing software MestReNova v.7.1.0–9185, both the 1D and the 2D data sets were overlaid. The 2D HSQC experiments were the most useful in identifying the major differences in the structure. Once a region of the molecule was identified that differed from the standard, additional NMR experiments were used to assign and confirm the new KmTx structures.

The structures of Abbotoxin and its 59-E-chloro-Abbotoxin were elucidated using similar techniques as those in Waters et al.,^8^ and the detailed data set is presented in the **Supporting information S1–S18**. These structures structures are the smallest congeners elucidated to date and differ significantly from KmTx2 in the C1-C18 region. The structures were five carbons fewer than KmTx2 all of which come from the polyol region. The HSQC overlay experiments showed that C23-C63 and C65–67 for KmTx2 are identical to C19–49 and C60–62 for the 59-E-chloro-Abbotoxin. The difference in the structures was the loss of C64, C13–14 and C16–17 (3 CH’s, 1 CH_2_ and 1 CH_3_ [68 amu]) from KmTx2. The key HMBC and COSY correlations and the deletion of carbon from KmTx2 for the final assembly of C1–18 of the chlorinated Abbotoxin are shown in the supporting information. Abbotoxin and its 59-E-chloro-Abbotoxin differ from each other only by the characteristic loss of a chlorine atom on the terminal diene. The relative configuration of these congeners was assigned by comparison to KmTx2 with careful analysis of NOE interactions for new stereogenic centers. For portions of the molecule where KmTx2 NMR overlay was too different for direct comparison, computational chemical shift calculations^2^ were carried out to confirm the relative configuration of new stereogenic centers and link them back to the known absolute configuration of C21 established in KmTx2 by degradation chemistry.^3^ Based on this method, the absolute configuration of Abbotoxin is 2S, 6*R*, 14S with C10 remaining ambiguous due to insufficient data to conclusively assign this sterocenter.

### HSQC Sterol Binding Experiments.

Standards were prepared by adding 2.0 mg (7.08 mM) KmTx8 in 200μL MeOD with 10μL CDCl_2_ to a 3 mm NMR tube. Helium was used to remove dissolved O_2_ and achieve cleaner NMR signals. HSQC and ROESY experiments were completed using a Varian 600 MHz NMR at 25°C. The HSQC data for Region II of the molecule (oxygenated carbons) is shown in blue above. In a separate NMR tube, 2.0 mg (7.08 mM) 8 in 200μL MeOD and 0.2 mg (2.46 mM) cholesterol in 10μL CDCl_2_ were combined and data acquired identically to the standard. The HSQC is shown in red above. The two data sets were then overlaid utilizing MestReNova 7.0.3–8830. This process was repeated using 0.3 mg cholesterol dissolved in CDCl_2_.

## Data Availability

Additional data supporting reported results can be provided by authors upon request. Toxin purification details from ARP, structure and NMR data from MTH, and black lipid studies from JR-F.
